# An Experimental Model: Modifications of the Chemical Silicone Medical Characteristics for an Improved Biocompatibility

**DOI:** 10.1007/s00266-026-05970-5

**Published:** 2026-08-27

**Authors:** Tian-Xi Li, Ming-Fei Lang, Di-Ya Su, Chen Zhang, Shi-Hui Wang, Xiao-Ying Dong, Cheng Han, Jie-Qing Wang, Jing Sun

**Affiliations:** 1 Plastic and Aesthetic Center, Dalian University Affiliated Xinhua Hospital, No. 156, Wansui Street, Shahekou District, Dalian, 116022 Liaoning China; 2 School of Basic Medical Sciences, Dalian University, No. 10, Xuefu Street, Jinzhou District, Dalian, 116622 Liaoning China; 3 College of Chemical and Environmental Engineering, Dalian University, No. 10, Xuefu Street, Jinzhou District, Dalian, 116622 Liaoning China

**Keywords:** Polydimethylsiloxane (PDMS), Polydopamine (PDA), Gelatin, Cell culture, Biocompatibility

## Abstract

**Objective:**

Despite its good biocompatibility and processability, the severe surface hydrophobicity of polydimethylsiloxane (PDMS) limits its biomedical applications. This study aimed to enhance the surface properties of PDMS by depositing gelatin onto it via polydopamine (PDA) mediation and to evaluate the resulting cell–material interactions.

**Methods and Results:**

The 1% gelatin-modified PDA-PDMS (G-PDA-PDMS) group showed the highest cell viability at 48 h. Surface hydrophilicity was significantly improved, with the contact angle reduced to 21.97° ± 2.06 (*p* <0.01). XPS confirmed successful modification, showing increased nitrogen signal and characteristic C=O/C-N peaks on G-PDA-PDMS. AFM revealed significantly higher surface roughness and altered nano-topography compared to PDMS (*p* <0.05). After interacting with cells, it was observed that the cell viability of G-PDA-PDMS was significantly higher than that of the other two groups (*p* <0.05). FITC/DAPI fluorescence staining showed that the number of cells in G-PDA-PDMS was higher than that in PDMS (*p* <0.05), and it exhibited a better cell spreading area (*p* <0.05). Calcein AM/PI fluorescence staining showed that the proportion of red fluorescence in PDA-PDMS and G-PDA-PDMS decreased significantly, while the green fluorescence increased significantly, indicating that they had a better cell survival rate compared with PDMS (*p* <0.05). The cell scratch test showed that the cell migration rate of G-PDA-PDMS was higher than that of PDA-PDMS (*p* <0.05). At the molecular level, G-PDA-PDMS significantly upregulated the mRNA expression of *TGF-β1*, *COL3A1*, *COL1A1*, and *α-SMA* (*p* <0.05), which was corroborated by increased *TGF-β1* protein production (*p* <0.05).

**Conclusion:**

Gelatin was successfully modified onto PDA-PDMS. The resulting G-PDA-PDMS material significantly improves surface hydrophilicity, enhances fibroblast proliferation and adhesion, and upregulates key extracellular matrix-related gene and protein expression, demonstrating excellent bioactivity and potential for biomedical applications.

**No Level Assigned:**

This journal requires that authors assign a level of evidence to each submission to which Evidence-Based Medicine rankings are applicable

## Introduction

Polydimethylsiloxane (PDMS) is widely used in many fields, such as architecture [1,2], microfluidics [3,4], chemistry [5,6], and biomedicine [7,8], due to its excellent optical transparency, biocompatibility, ease of fabrication, and cost-effectiveness. However, the intrinsic and strong hydrophobicity of its surface severely hampers initial cell adhesion and subsequent proliferation [9], which are fundamental to successful cell–material interactions [10]. This limitation necessitates effective surface modification to make PDMS suitable for biological applications.

Common modification strategies include physical treatments (e.g., plasma) and coatings with extracellular matrix (ECM) proteins. While simple, these approaches often yield transient or unstable surface properties [11]. Conversely, direct chemical modification can introduce complexity and risk leaving cytotoxic residues [12,13]. A robust and versatile alternative is the use of polydopamine (PDA), a bio-inspired coating that adheres to virtually any material under mild aqueous conditions [14]. The formed PDA layer not only enhances the surface hydrophilicity but also provides a reactive platform for the secondary immobilization of bioactive molecules via its catechol and amine groups. This method has proven effective in promoting the stable culture and function of various cell types, including mesenchymal stem cells, on PDMS [15,16]. In the experiment of Chuah et al. [16], coating a layer of polydopamine and another layer of adhesion protein on the PDMS substrate has been shown to improve adhesion proliferation and differentiation of MSC on its surface.

Agnieszka et al. [17] compared the effects of surface modification of PDMS materials with various proteins and found that collagen was the best. Given the high cost and batch variability associated with native ECM proteins like collagen, more affordable alternatives are sought. Gelatin, a hydrolyzed derivative of collagen, presents an excellent candidate. It is biocompatible, biodegradable, and retains critical cell-adhesive motifs such as the RGD sequence, making it beneficial for wound healing and tissue integration [18–20]. Gelatin establishes a stable bioactive interface with polydopamine (PDA)-coated surfaces via covalent bonding, primarily through nucleophilic attack by PDA amino groups (-NH₂) on EDC/NHS-activated carboxyl groups (-COOH) of gelatin [10,21]. This interaction enhances surface hydrophilicity and enables cell-binding motifs (e.g., RGD sequences) to directly regulate cellular adhesion and phenotypic transformation.

Building on the complementary advantages of PDA (universal adhesion) and gelatin (bioactivity and affordability), this study aimed to develop a gelatin-deposited PDA-PDMS (G-PDA-PDMS) substrate. We hypothesized that this modification would effectively overcome the inherent hydrophobicity of PDMS while imparting bioactive signals to direct fibroblast adhesion, proliferation, and ECM-related gene expression. This work provides a simple yet effective strategy for fabricating functional PDMS-based interfaces with potential applications in wound dressings and tissue engineering.

## Materials and Methods

### Reagents and Materials

Cell culture-related consumables, such as cell culture dishes and multi-well cell culture plates, all purchased from Sangon Biotech (China). The water used for preparing experimental reagents was ultrapure water. The remaining biological and chemical reagents are shown in Table 1.

**Table 1:** Reagents and materials

Categories	Vendor	Catalog Number
L929 (mouse fibroblasts)	HyCyte™, China	TCM-C749
Hydrochloric acid	Aladdin, China	H399826
Dopamine hydrochloride	Aladdin, China	D103111-25g
Tris (Hydroxymethyl) Aminomethane	Solarbio, China	T8060
SuperKine™ Maximum Sensitivity Cell Counting Kit-8 (CCK-8)	Abbkine, China	BMU106
TritonX-100	Meilunbio, China	MB2486
4% Paraformaldehyde Fixative Solution	Coolaber, China	SL1830-500mL
DAPI solution (1mg/mL)	Meilunbio, China	MA0127
FITC-Phallodin	MKbio, China	MX4403
0.25%Trypsin-EDTA, Phenol Red (modified)	Meilunbio, China	MA0233-1
PBS (10X)	Meilunbio, China	MA0016
Complete medium for L929	HyCyte™, China	TCM-G749
NcmSpin Cell/Tissue Total RNA Kit	NcmBiotech, China	M5105
RevertAid First Strand cDNA Synthesis Kit	Thermo Scientific™, USA	K1622
PowerUp™ SYBR™ Green Master Mix	Thermo Scientific™, USA	A25742
Mouse TGF-*β*1 ELISA Kit	BOSTER, USA	EK0515

### Preparation of PDMS Substrates

PDMS substrates were prepared by thoroughly mixing the base and curing agent (Metu High-Tech Materials Group) at a 10:1 (W/W) ratio. The mixture was poured onto polystyrene plates, degassed in a vacuum oven for 60 min to remove air bubbles, and then cured at 70 ℃ for 120 min.

### Surface Modification with PDA

A 0.2% (W/V%) polydopamine solution was prepared by dissolving 200 mg of dopamine hydrochloride in 100 mL of 10 mM Tris–HCl buffer (pH adjusted to 8.5 using 1 M HCl). PDMS substrates were first treated with ozone/UV for 30 min and then immersed in the freshly prepared PDA solution for 24 h at room temperature. The resulting PDA-coated PDMS (PDA-PDMS) samples were rinsed twice with deionized water and dried under a gentle stream of nitrogen.

### Gelatin Deposition onto PDA-PDMS

PDA-PDMS samples were activated by immersion in a cold 2-morpholinoethanesulfonic acid (MES) buffer solution (pH 5.5) containing 50 mM EDC (0.958 g) and 50 mM NHS (0.575 g) for 15 min. After activation, samples were rinsed with PBS to remove excess EDC/NHS. Separately, gelatin solutions at concentrations of 0.5%, 1%, and 2% (w/v) were prepared in PBS at 40 ℃ until clear. The activated PDA-PDMS samples were then immersed in the respective gelatin solutions and incubated overnight at 37 ℃. Following incubation, samples were rinsed twice with PBS and air-dried, yielding the final materials designated as 0.5G-PDA-PDMS, 1G-PDA-PDMS, and 2G-PDA-PDMS.

### Determination of Optimal Gelatin Concentration (CCK-8 Assay)

Mouse fibroblast L929 cells were seeded onto PDMS, PDA-PDMS, and the three gelatin-grafted variants at a density of 8×10^3^ cells/well in 96-well plates (n=3). After 48 h of culture in complete medium at 37 ℃ with 5% CO_2_, cell viability was assessed using the CCK-8 kit according to the manufacturer’s instructions (Tecan, Switzerland). Absorbance was measured at 450 nm. The 1% gelatin formulation (1G-PDA-PDMS, subsequently abbreviated as G-PDA-PDMS) demonstrated the highest cell viability and was therefore selected for all subsequent experiments.

$$\begin{gathered} {\text{Cell Viability}} = \left( {\frac{{OD_{{{\mathrm{group1}}}} - OD_{{{\mathrm{group3}}}} }}{{OD_{{{\mathrm{group2}}}} - OD_{{{\mathrm{group3}}}} }}} \right) \times 100\% \hfill \\ \left( {\text{group1:exprimental group, group2: PDMS group, group3: blank group}} \right) \hfill \\ \end{gathered}$$

### Surface Characterization

#### Water Contact Angle

The static water contact angles of PDMS, PDA-PDMS, and G-PDA-PDMS samples were measured using a contact angle goniometer (JY-PHb, Jinhe Instrument, China). A 2-µL deionized water droplet was dispensed onto the sample surface, and the contact angle was determined using the tangent method. Three random locations were measured for each sample, and the average value was calculated.

#### X-ray Photoelectron Spectroscopy (XPS)

The surface chemical composition of PDMS, PDA-PDMS, and G-PDA-PDMS was analyzed using a Thermo Fisher Scientific K-Alpha spectrometer with a monochromatic Al K*α* X-ray source. Survey scans and high-resolution spectra for C1s, O1s, Si2p, and N1s were collected.

#### Atomic Force Microscopy (AFM)

The surface topography of the samples was examined using a Bruker Dimension Icon AFM in tapping mode. Samples were fixed on glass slides, and scans were performed over a 2 µm × 2 µm area.

### Cell Culture

Mouse fibroblast L929 cells were maintained in complete growth medium at 37 ℃, 5% CO_2_. Cells between passages 3 and 5 were used for all experiments.

### Evaluation of Cell–Material Interactions

#### CCK-8 Assay

Cells were seeded at 8×10^3^ cells/well in 96-well plates (n=3) and cultured for 24 h, 48 h, and 72 h. Viability was assessed via CCK-8 assay as described in “Determination of Optimal Gelatin Concentration (CCK-8 Assay)” Section.

#### FITC/DAPI Staining

Cells were seeded at 4×10^4^ cells/well in 24-well plates (n=3). After 24 h, cells were fixed with 4% paraformaldehyde, permeabilized, and stained with FITC-phalloidin (for actin cytoskeleton) and DAPI (for nuclei). Images were captured using fluorescence microscopy (Nikon, Japan). Cell number and individual cell spreading area were quantified using ImageJ software. Relative spreading area was calculated as follows:

$$\begin{gathered} {\text{Relative Spreading Area}} = \frac{{{\text{Total Cell Spreading Area}}_{{{\mathrm{group}}}} /{\text{Cell count}}_{{{\mathrm{group}}}} }}{{{\text{Single Cell Spreading Area}}_{{{\mathrm{PDMS}}}} }} \times 100\% \hfill \\ \left( {\text{group = PDMS/PDA - PDMS/G - PDA - PDMS}} \right) \hfill \\ \end{gathered}$$

#### Calcein AM/PI Staining

Cells were seeded as above (4×10^4^ cells/well in 24-well plates, n=3). After 24 h, samples were incubated with Calcein AM and PI working solution for 30 min. Images were captured using fluorescence microscopy (Nikon, Japan). Live (green) and dead (red) cells were visualized and counted to calculate cell survival rate.

$$Cell\;Survival\;Rate = \frac{{N_{{live,group}} }}{{N_{{total,group}} }}{\text{(where N total includes both live and dead cells, group = PDMS/PDA - PDMS/G - PDA - PDMS)}}$$

$$\begin{gathered} {\text{Relative Survival Rate}}_{{{\mathrm{group}}}} { = }\frac{{{\text{Survival Rate}}_{{{\mathrm{group}}}} }}{{{\text{Survival Rate}}_{{{\mathrm{PDMS}}}} }} \hfill \\ \left( {\text{group = PDMS/PDA - PDMS/G - PDA - PDMS}} \right) \hfill \\ \end{gathered}$$

#### Scratch Wound Assay

Cells were seeded at 40×10^4^ cells/well in 6-well plates (n=3) and grown to confluence. A scratch was created using a sterile pipette tip. Cells were then cultured in low-serum medium (2% HS). The scratch width was measured at 0 h, 18 h, and 36 h under a microscope (Olympus, Japan). The migration rate was calculated as follows:

$$Migration\;Rate = \frac{{Scratch\;width_{{0h}} - Scratch\;width_{t} }}{{Scratch\;width_{{0h}} }} \times 100\% (where\;t = 18\;h\;or\;36\;h)$$

### Gene Expression Analysis (RT-qPCR)

L929 cells were seeded on the materials at 4×10^4^ cells/well (n=3) and cultured for 24 h, 36 h, and 48 h. Total RNA was extracted using NcmSpin Cell/Tissue Total RNA Kit, and purity was verified (A260/A280 > 1.8). cDNA was synthesized with a RevertAid First Strand cDNA Synthesis Kit. RT-qPCR was performed using PowerUp™ SYBR™ Green Master Mix on a CFX connect system (BIO-RAD, China) with primers listed in Table 2. Glyceraldehyde-3-phosphate dehydrogenase (GAPDH) served as the endogenous control. The relative mRNA expression levels of transforming growth factor-*β*1 (*TGF-β1*), collagen type I alpha 1 chain (*COL1A1*), collagen type III alpha 1 chain (*COL3A1*), and alpha-smooth muscle actin (*α-SMA*) were analyzed.

**Table 2:** Specific primers

Primers name		Primers sequence(5'–3')
*GAPDH*	Forward Primer	GGTGAAAGGTCGGTGTGAACG
	Reverse Primer	CTCGCTCCTGGAAGATGGTG
*TGF-β1*	Forward Primer	GCATTGGCAAAGGTCGGTTT
	Reverse Primer	TGCCTCTCGGAACCATGAAC
*COL3A1*	Forward Primer	TGACTGTCCCACGTAAGCAC
	Reverse Primer	AGGAAGATCAGGAGGGCCAT
*COL1A1*	Forward Primer	CCCAGTGGCGGTTATGACTT
	Reverse Primer	CTCAAGGTCACGGTCACGAA
*α-SMA*	Forward Primer	GTCCCAGACATCAGGGAGTAA
	Reverse Primer	TCGGATACTTCAGCGTCAGGA

### Protein Production Analysis (ELISA)

L929 cells were seeded as for ELISA. After 24 h, the medium was replaced with fresh MEM, and the conditioned medium was collected after a further 24 h, 36 h, and 48 h of culture. The concentration of secreted *TGF-β1* protein in the supernatant was quantified using a ELISA kit according to the manufacturer's instructions (Tecan, Switzerland). Absorbance was measured at 450 nm. A standard curve with an R^2^ value≥0.99 was used for quantification. Due to poor cell adhesion on unmodified PDMS, which compromised supernatant collection for this group, the ELISA data were compared only between the PDA-PDMS and G-PDA-PDMS groups.

### Statistical Analysis

All quantitative data are presented as mean ± standard deviation (SD) from at least three independent experiments. Statistical analyses were performed using GraphPad Prism 10 software. Comparisons between two groups were made using Student’s *t*-test. For comparisons among more than two groups, one-way analysis of variance (ANOVA) followed by Tukey’s post hoc test was applied. The difference was considered statistically significant when *p* <0.05.

## Results

### Determination of Optimal Gelatin Concentration

**Fig. 1:**
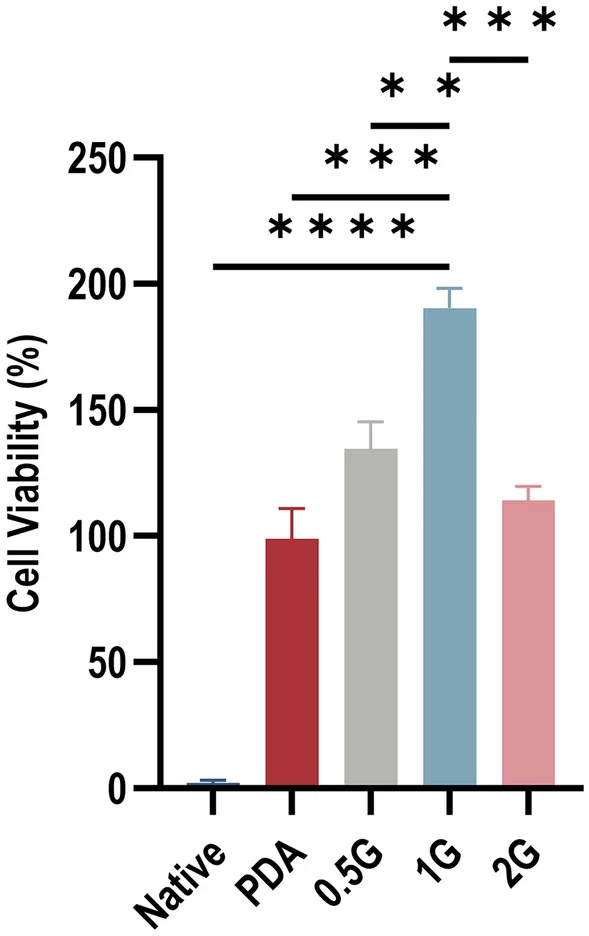
Cell viability following interaction with PDMS materials modified with different gelatin concentrations. (Native: native PDMS; PDA: PDMS modified with PDA; 0.5G, 1G, 2G: PDA-PDMS further modified with 0.5%, 1%, and 2% gelatin, respectively. (**p <0.01, ***p <0.001, ****p <0.0001.)

The CCK-8 assay revealed that all modified surfaces enhanced the cell viability compared to pristine PDMS (control). The 1G-PDA-PDMS group exhibited the highest cell viability at 190.25% ± 7.99 (*p* <0.0001, Fig. 1). Consequently, the 1% gelatin concentration was selected for all subsequent experiments, and the material is hereafter referred to as G-PDA-PDMS.

### Surface Wettability

**Fig. 2:**
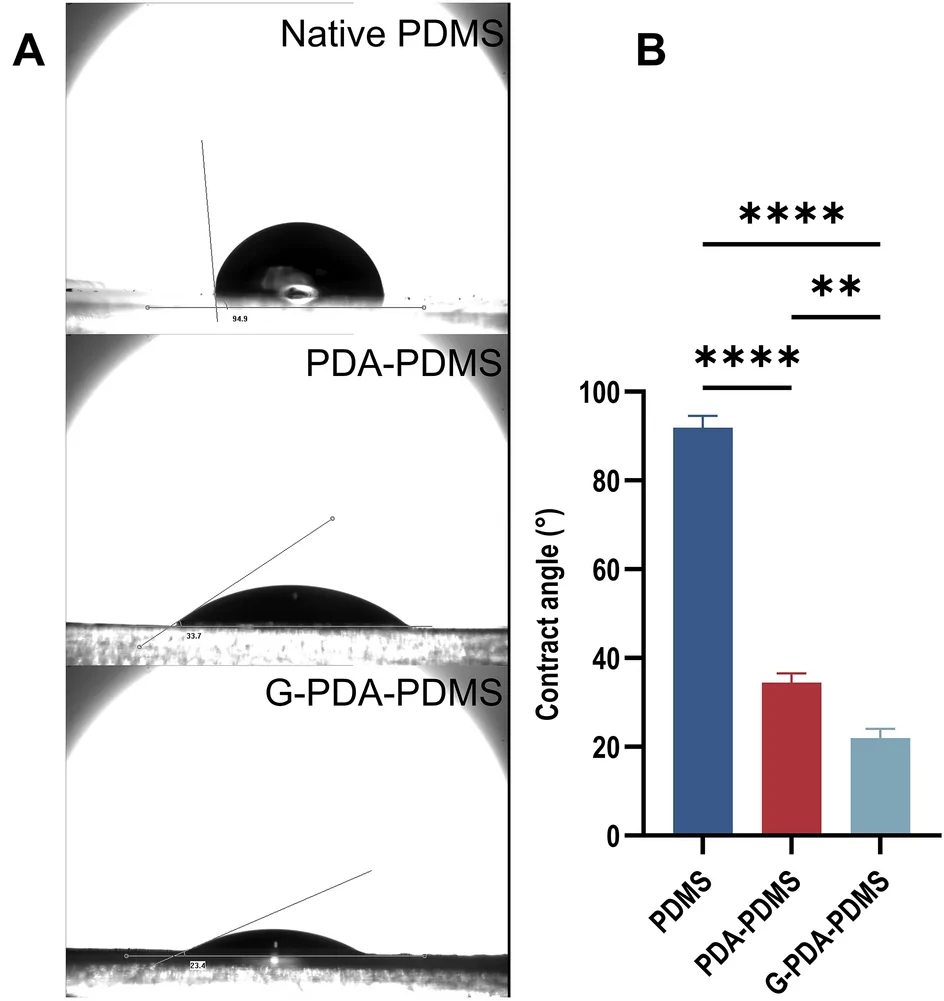
**A** Contact angles of different materials; **B** Quantitative analysis. (**p <0.01, ****p <0.0001.)

Surface modification significantly altered the material wettability. The average water contact angle of pristine PDMS was 91.9° ± 2.61. This angle markedly decreased to 34.47° ± 2.06 for PDA-PDMS and further to 21.97° ± 2.06 for G-PDA-PDMS (*p* <0.0001 for both vs. PDMS, Fig. 2B), indicating a substantial enhancement in surface hydrophilicity.

### XPS Analysis

**Fig. 3:**
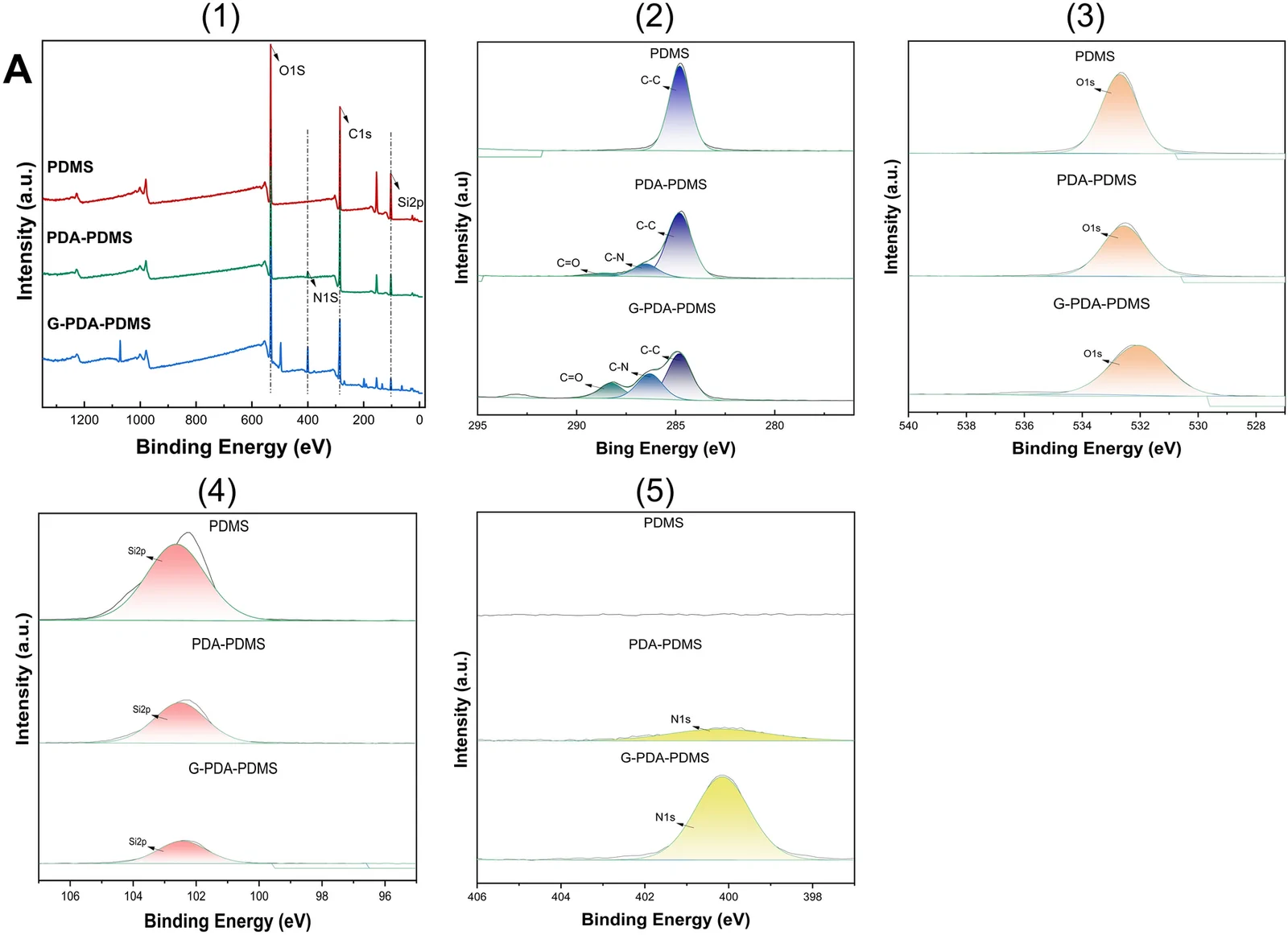
**A** Qualitative and quantitative analysis of the full spectrum (1) and the fine spectra of C1s (2), O1s (3), Si2p (4), and N1s (5) by XPS analysis

XPS high-resolution C1s spectra were deconvoluted to identify carbon chemical states. All groups showed a peak at 284.55 eV, corresponding to C-C/C-H bonds. Notably, the G-PDA-PDMS spectrum displayed two additional peaks: one at 285.87 eV (C=O, from carboxyl/amide groups) and another at 288.89 eV (C–N bonds). This is consistent with the research results of Elke et al. [22]. Furthermore, a distinct N1s peak was detected only on the G-PDA-PDMS surface, confirming successful gelatin grafting. In the study by Haejin et al.[23], the nitrogen peak belonging to primary amines at the N1s binding-energy sites can also be observed. The intensities of the O1s and Si2p peaks were lower in G-PDA-PDMS compared to pristine PDMS in Fig. 3.

### AFM

**Fig. 4:**
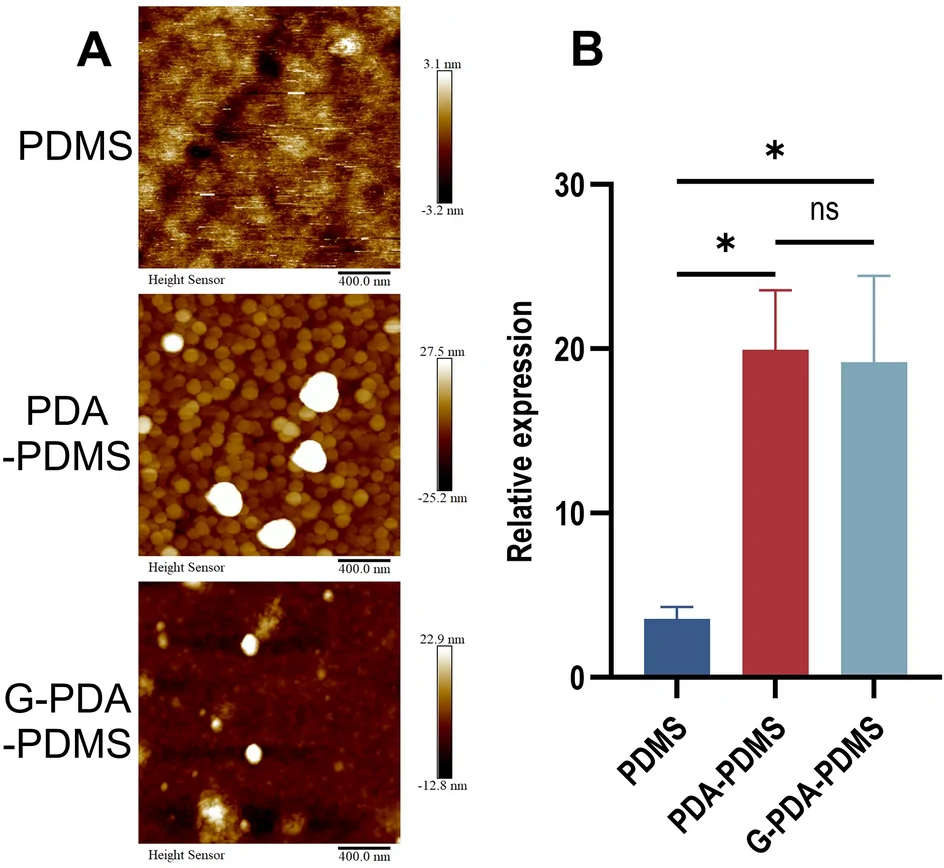
**A** 2D surface nano-topography and surface roughness of each material; **B** Quantitative analysis of the surface roughness of each material. (ns indicates *p* >0.05, *p <0.05.)

AFM revealed distinct nanoscale surface morphologies. In Fig. 4, pristine PDMS had a smooth surface with a roughness of 3.57 ± 0.72 nm. PDA coating introduced nanoparticle or island-like aggregates, significantly increasing the roughness to 19.95 ± 3.61 nm (*p* <0.05 vs. PDMS). Subsequent gelatin grafting (G-PDA-PDMS) yielded a more uniform and flattened surface, with a roughness of 19.2 ± 5.25 nm, which was not significantly different from that of PDA-PDMS alone.

### Cell Viability Over Time

**Fig. 5:**
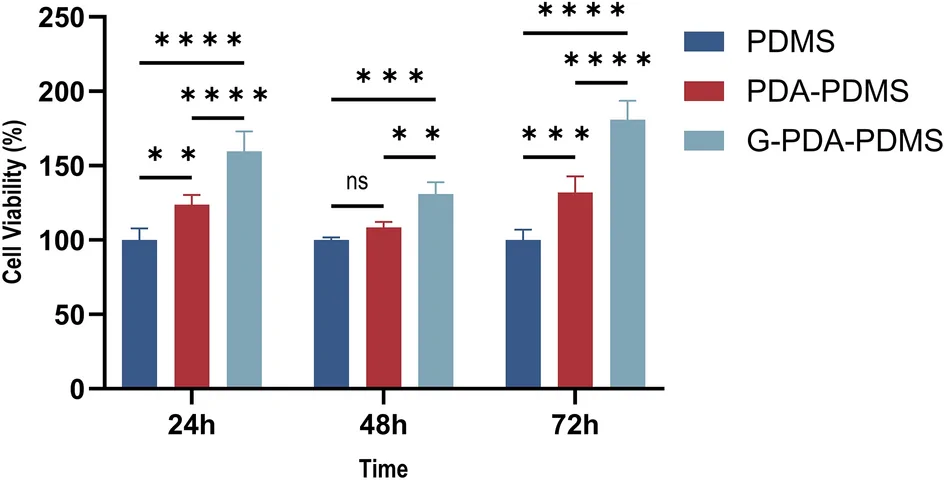
Quantitative analysis of cell viability after the interaction of each material with cells for 24 h, 48 h, and 72 h. (ns indicates p>0.05, **p <0.01, ***p <0.001, ****p <0.0001.)

A time-course CCK-8 assay over 72 h demonstrated superior performance of G-PDA-PDMS. Cell viability on G-PDA-PDMS was significantly higher than on other groups at all time points: 159.67% ± 13.32 at 24 h (*p* <0.0001), 131% ± 7.79 at 48 h (*p* <0.001), and 181% ± 12.68 at 72 h (*p* <0.0001), as shown in Fig. 5. The viability on G-PDA-PDMS was also significantly higher than on PDA-PDMS at these time points (*p* <0.01).

### Cell Adhesion and Spreading

**Fig. 6:**
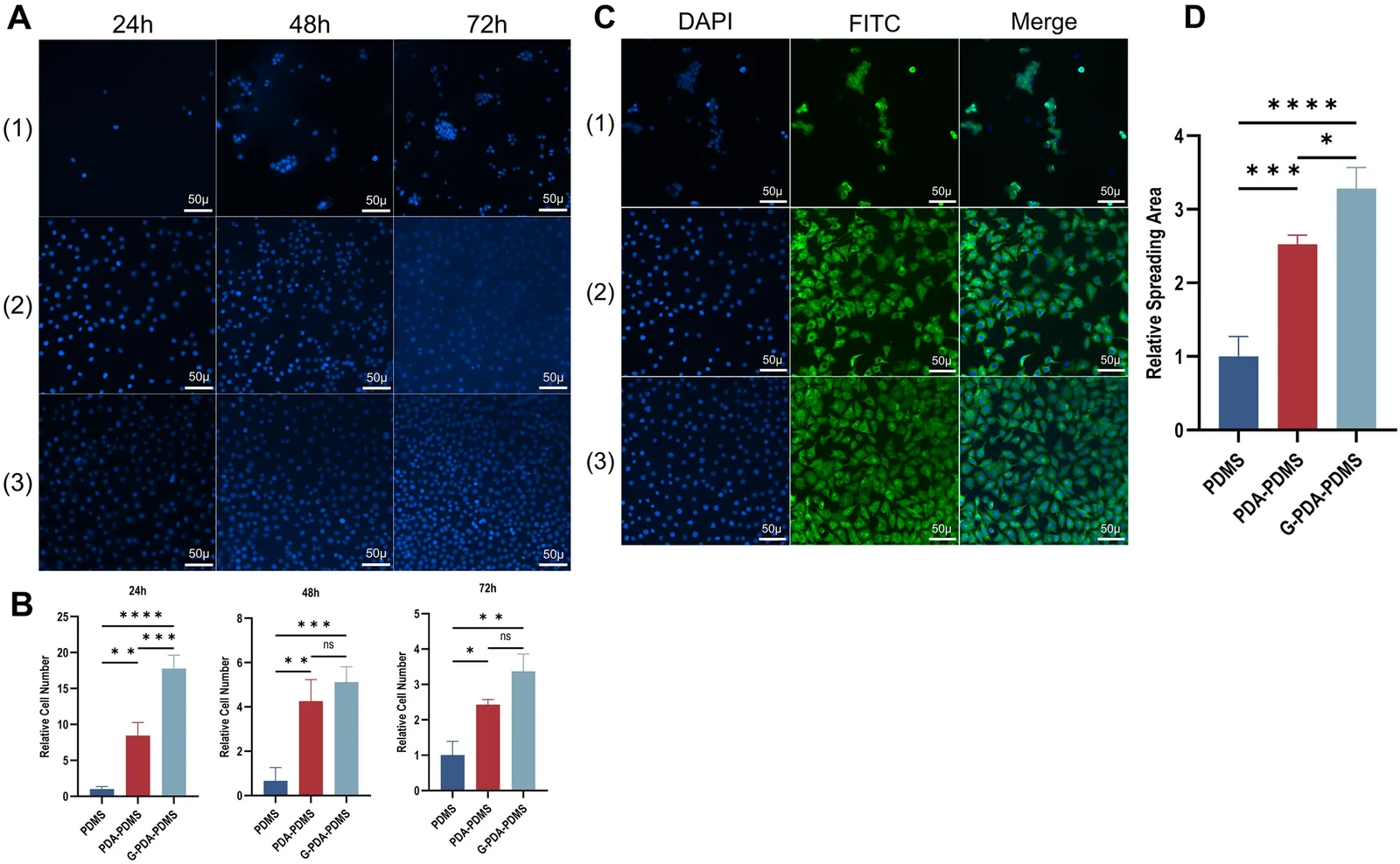
**A** DAPI staining images showing L929 cell nuclei after 24 h (1), 48 h (2), and 72 h (3) of interacting with the indicated materials. **B** Quantification of cell numbers corresponding to panel A (statistical significance is denoted as described in previous figure captions). **C** Representative fluorescence images of cells stained with FITC-phalloidin (actin cytoskeleton) and DAPI (nuclei) after 48 h of culture on PDMS (1), PDA-PDMS (2), and G-PDA-PDMS (3). **D** Quantification of relative single-cell spreading area derived from panel C. (ns indicates *p* >0.05, *p <0.05, **p <0.01, ***p <0.001, ****p <0.0001.)

FITC/DAPI staining quantified cell adhesion and morphology after 24 h. The number of cells adhered to G-PDA-PDMS was 17.75 times ± 1.88 that on PDMS (*p* <0.0001). The relative spreading area of individual cells on G-PDA-PDMS was 3.28 times ± 0.29 greater than on PDMS (*p* <0.0001) and was also significantly better than on PDA-PDMS (*p* <0.05) in Fig. 6.

### Cytocompatibility

**Fig. 7:**
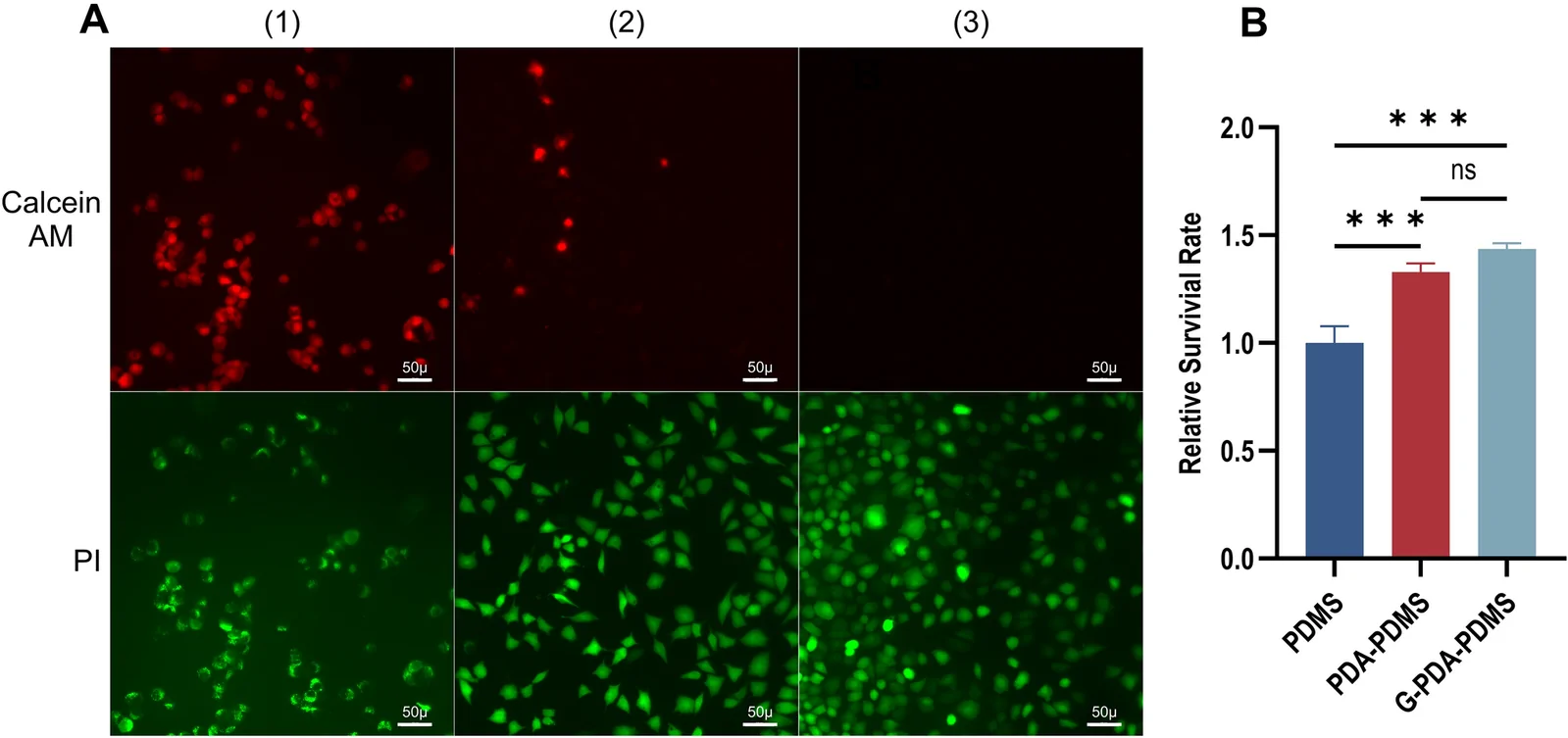
**A** Calcein AM/PI staining images after the interaction of cells with PDMS (1), PDA-PDMS (2), and G-PDA-PDMS (3). **B** Quantitative analysis of relative cell viability. (ns indicates *p* >0.05, ***p <0.001)

Calcein AM/PI staining after 24-hour culture indicated excellent cytocompatibility. G-PDA-PDMS surfaces showed prevalent green fluorescence (live cells) and negligible red fluorescence (dead cells), whereas pristine PDMS showed the opposite trend. Quantitative analysis revealed that the cell survival rates on PDA-PDMS and G-PDA-PDMS were 1.33 times ± 0.04 and 1.44 times ± 0.03 that of the PDMS control, respectively (*p* <0.05) in Fig. 7.

### Cell Migration

**Fig. 8:**
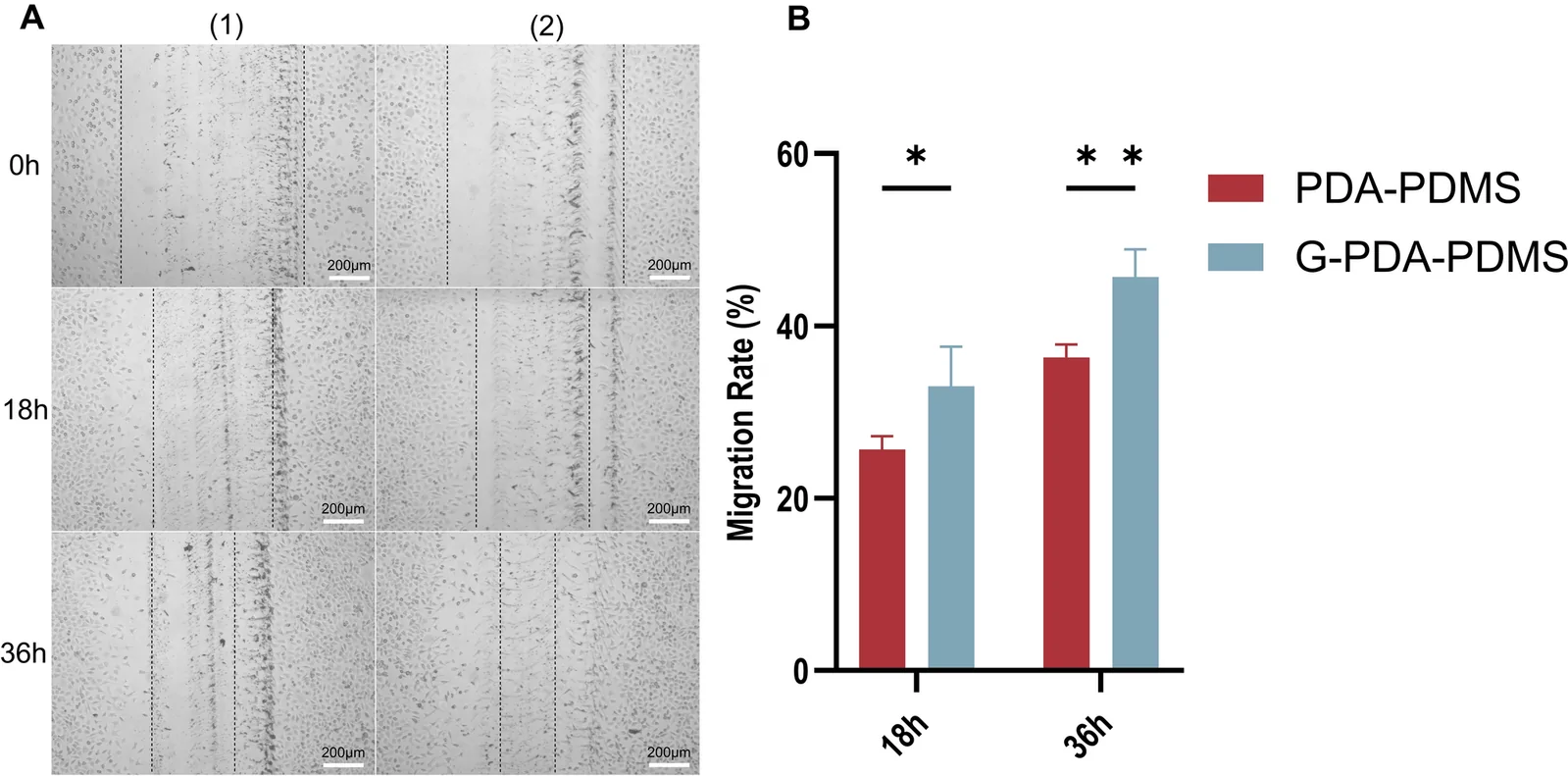
**A** Images of cell scratches after the interaction between PDA-PDMS, G-PDA-PDMS, and cells. **B** Quantitative analysis of cell migration rate. (*p <0.05, **p <0.01.)

The scratch wound healing assay demonstrated enhanced cell migration on modified surfaces. At 18 h, the migration rate was 25.67% ± 5.19 for PDA-PDMS and significantly higher for G-PDA-PDMS. By 36 h, the migration rate reached 36.33% ± 5.19 for PDA-PDMS, and was again significantly greater for G-PDA-PDMS (*p* <0.05 for both time points) in Fig. 8.

### Gene Expression Analysis (RT-qPCR)

**Fig. 9:**
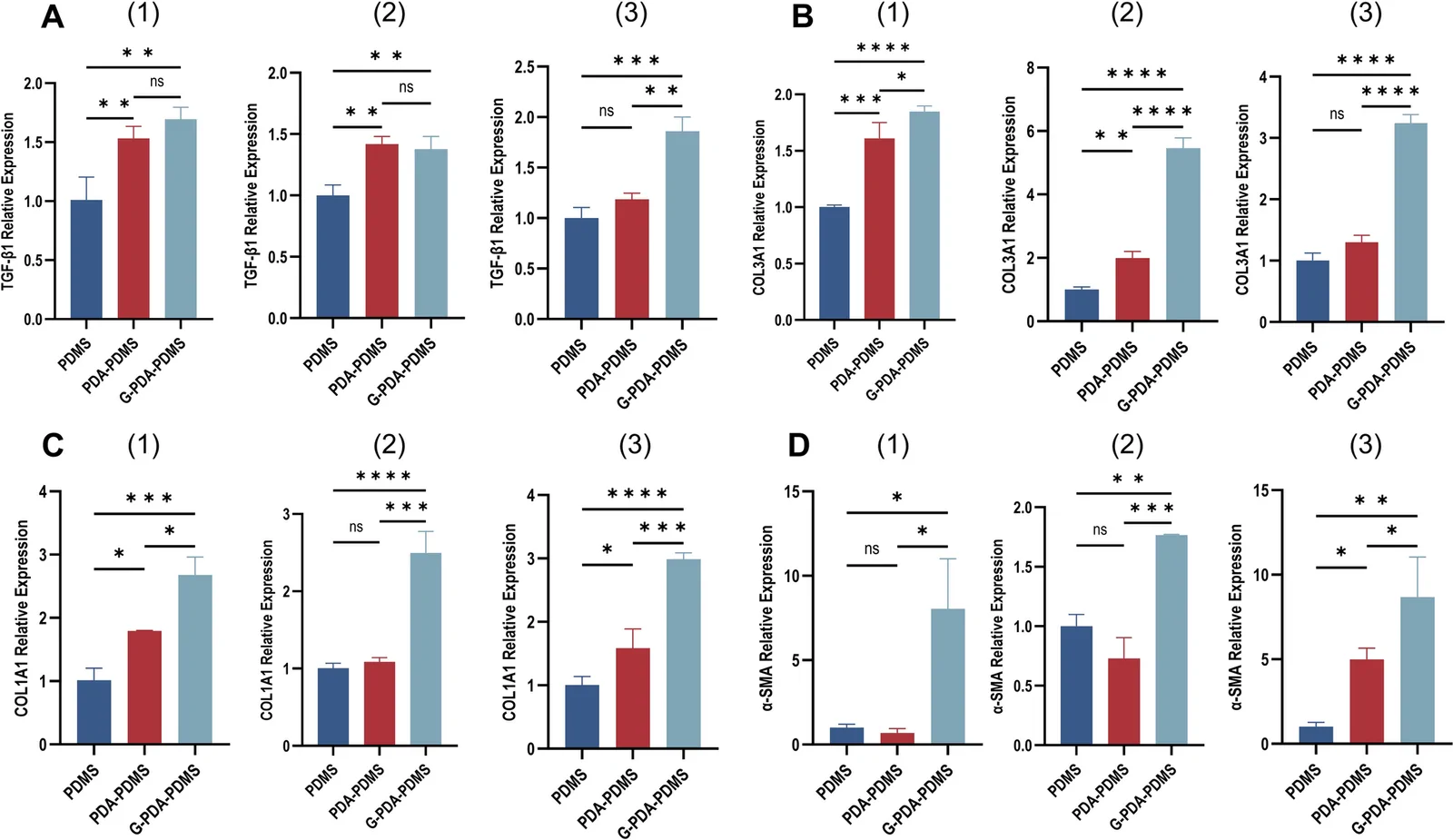
**A** Relative mRNA expression of *TGF-β1* in L929 cells cultured on different materials for 24 h (1), 36 h (2), and 48 h (3). **B** Relative mRNA expression of *COL3A1* after 24 h (1), 36 h (2), and 48 h (3) of cell–material interaction. **C** Relative mRNA expression of *COL1A1* following 24 h (1), 36 h (2), and 48 h (3) of culture. **D** Relative mRNA expression of *α-SMA* after 24 h (1), 36 h (2), and 48 h (3) of interaction with the materials. (ns indicates *p* >0.05, *p <0.05, **p <0.01, ***p <0.001, ****p <0.0001.)

RT-qPCR assessed the expression of extracellular matrix-related genes (Fig. 9). Compared to the control (PDMS), G-PDA-PDMS significantly upregulated all target genes. The expression of *TGF-β1* was 1.69-fold (24 h, *p* <0.01), 1.38-fold (36 h, *p* <0.05), and 1.86-fold (48 h, *p* <0.001) higher. The expression of *COL3A1* was elevated by 1.85-fold (24 h), 5.46-fold (36 h), and 3.24-fold (48 h) (*p* <0.05 for all). The expression of *COL1A1* increased by 2.68-fold (24 h), 2.50-fold (36 h), and 2.99-fold (48 h) (*p* <0.05 for all). The expression of *α-SMA* was markedly upregulated by 8.04-fold (24 h), 1.77-fold (36 h), and 8.68-fold (48 h) (*p* <0.05 for all). At most time points, the expression levels on G-PDA-PDMS were also significantly higher than those on PDA-PDMS.

### TGF-β1 Protein Production (ELISA)

**Fig. 10:**
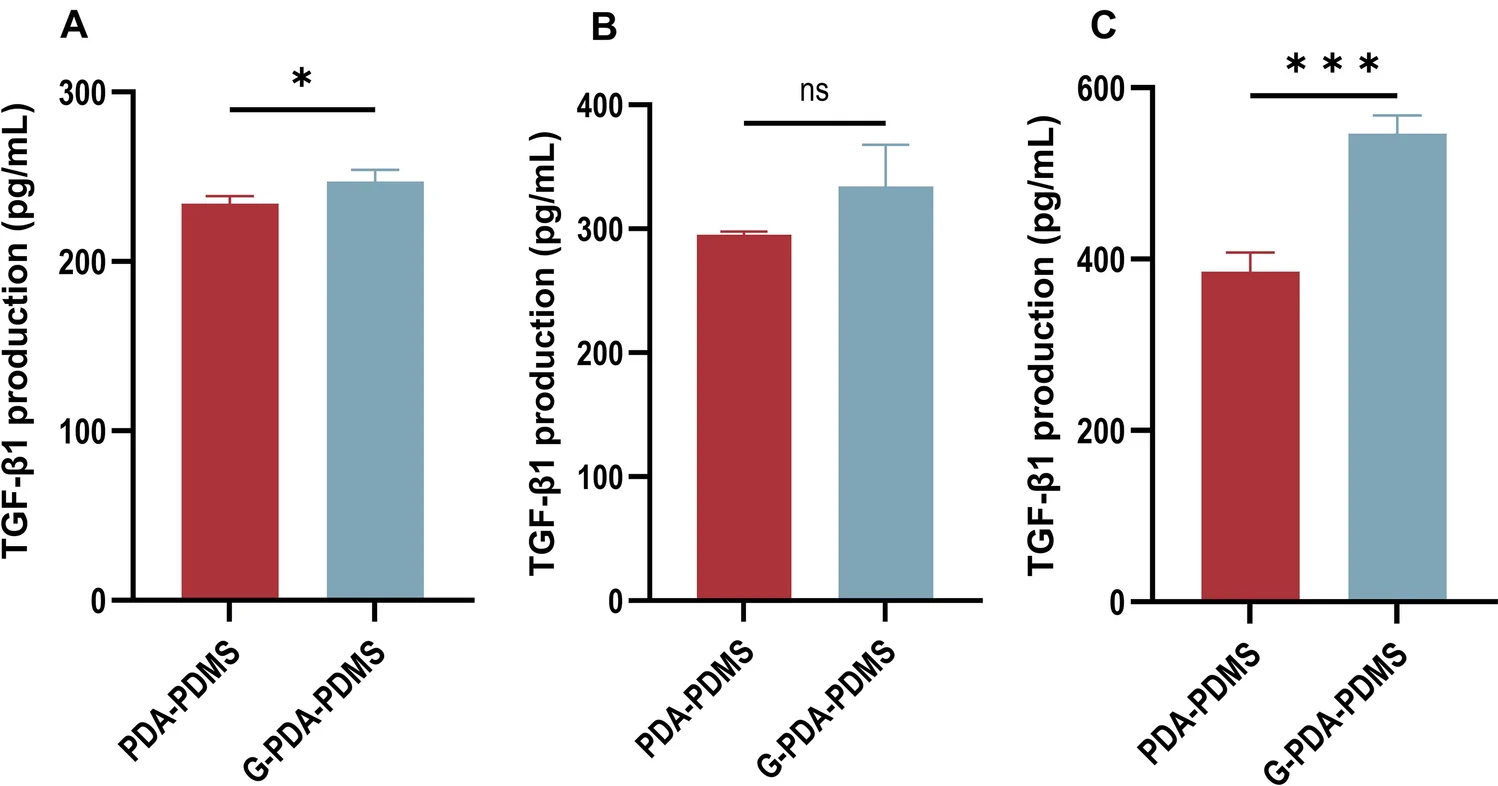
*TGF-β1* protein production levels measured by ELISA after 24 h (**A**), 36 h (**B**), and 48 h (**C**) of cell–material interaction with PDA-PDMS and G-PDA-PDMS. (ns indicates* p* >0.05, *p <0.05, ***p <0.001.)

The production of *TGF-β1* in the cell supernatant was detected after the cells interacted with the materials for 24 h, 36 h, and 48 h. The experimental results showed that after 24 h, the increase in *TGF-β1* in the G-PDA-PDMS group was 274.21 pg/mL ± 6.8 and that in the PDA-PDMS group was 234.05 pg/mL ± 4.58 (*p* <0.05). After 36 h, the increase in *TGF-β1* in the G-PDA-PDMS group was 334.17 pg/mL and that in the PDA-PDMS group was 295.27 pg/mL (*p* >0.05). After 48 h, the increase in *TGF-β1* in the G-PDA-PDMS group was 546.24 pg/mL ± 21.31 and that in the PDA-PDMS group was 385.20 pg/mL ± 22.61 (*p* <0.001). At 36 h and 48 h, it could be seen that the trend and significance of *TGF-β1* secretion were consistent with the relative expression of *TGF-β1* detected by RT-qPCR in Fig. 10.

### Statistical analysis

To provide an overview of the surface properties and cellular responses across the three material groups, key quantitative data are summarized in Table 3.

**Table 3:** Statistical analysis summary (PDA: PDMS modified with PDA, G: PDA-PDMS further modified with gelatin)

Parameter	PDMS	PDA-PDMS	G-PDA-PDMS	Statistical Significance
Water contact angle (°)	91.9° ± 2.61	34.47° ± 2.06	21.97° ± 2.06	*p* < 0.0001 (both vs. PDMS)
Roughness (nm)	3.57 ± 0.72	19.95 ± 3.61	19.2 ± 5.25	*p* < 0.05 (PDA vs. PDMS); ns (G vs. PDA)
Cell viability (24 h)	100.00% ± 7.79	123.75% ± 6.55	159.67 % ± 13.32	*p* < 0.0001 (G vs. PDMS); *p* < 0.0001 (PDA vs. G)
Relative cell number (24 h)	1.00 times ± 0.35	8.45 times ± 1.81	17.75 times ± 1.88	*p* < 0.0001 (G vs. PDMS); *p* < 0.001 (PDA vs. G)
Relative spreading area (24 h)	1.00 times ± 0.27	2.53 times ± 0.12	3.28 times ± 0.29	*p* < 0.0001 (G vs. PDMS); *p* < 0.05 (PDA vs. G)
Relative survival rate	1.00 times ± 0.08	1.33 times ± 0.04	1.44 times ± 0.03	*p* < 0.001 (G vs. PDMS)
Migration rate (36 h)	-	36.33 % ± 1.53	45.67 % ± 3.21	*p* < 0.01 (G vs. PDA)
*TGF-β1 mRNA* (48 h)	1.00 times ± 0.11	1.19 times ± 0.06	1.86 times ± 0.14	*p* < 0.001 (G vs. PDMS); *p* < 0.01 (PDA vs. G)
*COL3A1 mRNA* (48 h)	1.01 times ± 0.12	1.30 times ± 0.12	3.24 times ± 0.14	*p* < 0.0001 (G vs. PDMS and PDA vs. G)
*COL1A1 mRNA* (48 h)	1.01 times ± 0.13	1.59 times ± 0.3	2.99 times ± 0.11	*p* < 0.0001 (G vs. PDMS); *p* < 0.001 (PDA vs. G)
*α-SMA mRNA* (48 h)	1.02 times ± 0.25	5.46 times ± 0.32	8.68 times ± 2.37	*p* < 0.01 (G vs. PDMS); *p* < 0.05 (PDA vs. G)
*TGF-β1 protein*	-	385.20 pg/mL ± 22.61	546.24 pg/mL ± 21.31	*p* < 0.001 (PDA vs. G)

## Discussion

This study successfully developed a biofunctional PDMS interface through sequential surface modification with polydopamine (PDA) and gelatin. The modification strategy effectively addressed the intrinsic hydrophobicity of PDMS and imparted bioactive cues, leading to significantly enhanced fibroblast adhesion, proliferation, migration, and pro-healing gene expression.

The biological efficacy of surface modification demonstrated a nonlinear relationship with coating concentration. For the primary PDA layer, a 0.2% (W/V%) concentration was optimal. Lower concentrations may result in insufficient deposition and inadequate presentation of active sites, while higher concentrations could lead to overly thick or unstable PDA layers, potentially masking sites or introducing cytotoxicity from residual monomers [24]. CCK-8 and live/dead staining results confirmed the superiority of 0.2% PDA-PDMS in supporting cell proliferation and viability. Subsequent grafting of gelatin onto the optimized PDA-PDMS surface via EDC/NHS chemistry revealed that a 1% (W/V%) gelatin concentration yielded the highest cell viability. A lower concentration likely provides an insufficient density of cell-adhesive motifs (e.g., RGD sequences), failing to effectively promote cell interactions [25,26]. Conversely, a higher concentration may form a hydrogel layer that acts as a physical barrier, hindering direct cell contact with the underlying material and potentially impeding nutrient diffusion [27]. Furthermore, excessively high RGD density has been reported to paradoxically inhibit cell attachment and proliferation in other systems [28–30], which may explain the suboptimal performance of the 2% gelatin group. Therefore, the 1% gelatin-PDA formulation represents the most effective concentration for promoting fibroblast interactions on PDMS [31].

The strong hydrophobicity of pristine PDMS is a major barrier for biomedical applications. PDA serves as a versatile “molecular glue” through a dual mechanism. First, its catechol and amine groups enable self-polymerization and robust adhesion to the PDMS surface under mild alkaline conditions, forming a stable, hydrophilic intermediate layer. XPS analysis, evidenced by new C=O/C-N peaks in the C1s spectrum and a distinct N1s signal, confirmed the successful deposition of PDA and the introduction of polar functional groups, which are responsible for the dramatic reduction in water contact angle. Second, the reactive quinone groups on the PDA coating allow for the covalent grafting of gelatin via Schiff base or Michael addition reactions [32]. Gelatin, a collagen derivative rich in RGD sequences, is inherently hydrophilic. The synergistic effect of this dual modification endowed G-PDA-PDMS with optimal wettability (the lowest contact angle). AFM revealed that surface topography evolved in tandem with chemistry. PDA deposition introduced nanoparticle aggregates, significantly increasing surface roughness compared to smooth PDMS. Subsequent gelatin grafting modulated this topography, resulting in a more uniform surface for G-PDA-PDMS while maintaining elevated roughness. This engineered nano-topography, combined with the bioactive chemical signals, is crucial for directing cell response.

Consistent with improved surface properties, G-PDA-PDMS demonstrated superior cytocompatibility and bioactivity across multiple phenotypic assays. Calcein AM/PI staining indicated minimal cytotoxicity, with significantly higher cell survival rates compared to unmodified PDMS. Quantitative analysis of FITC/DAPI images confirmed that G-PDA-PDMS supported the greatest cell adhesion density and the largest individual cell spreading area. In scratch assay, cells on G-PDA-PDMS exhibited the highest migration rate. These results collectively underscore that the gelatin-PDA modification significantly promotes key wound healing phenotypes—proliferation, adhesion, spreading, and migration—compared to PDMS or PDA modification alone. This study aligns with the established principle that surface wettability is a primary determinant of cell adhesion and proliferation, with moderate hydrophilicity providing the optimal interface [33].

The most compelling evidence of bioactivity comes from molecular analyses. RT-qPCR revealed that G-PDA-PDMS significantly upregulated the expression of key genes involved in wound healing: transforming growth factor-β1 (*TGF-β1*, a master regulator of fibroblast activity), collagen type I alpha 1 chain (*COL1A1*) and collagen type III alpha 1 chain (*COL3A1*, core extracellular matrix components), and alpha-smooth muscle actin (*α-SMA*, associated with contractile function). *TGF-β1* is a core factor regulating fibroblast proliferation, differentiation into myofibroblasts, and extracellular matrix synthesis. Type III and type I collagen are core extracellular matrix components for granulation tissue formation and wound remodeling. The upregulation of these genes indicates that this material can effectively promote fibroblasts to synthesize and deposit extracellular matrix, thereby accelerating wound filling and the recovery of tissue strength [34]. The significant increase in *α-SMA*, a hallmark of myofibroblast differentiation [35], suggests G-PDA-PDMS can drive this functional transition essential for wound contraction. ELISA confirmed these findings at the protein level, showing increased *TGF-β1* secretion [36]. The upregulation of these factors indicates that the material stimulates fibroblasts to secrete key signaling molecules for wound healing, which could facilitate subsequent stages of healing, such as angiogenesis and tissue regeneration. The extensive activation at the gene level is consistent with the observed phenotypic enhancements, such as rapid cell proliferation, strong migratory capacity, and improved spreading morphology. These results provide preliminary molecular insights into the potential application of this material in wound healing. We hypothesize that the RGD sequences in the grafted gelatin activate downstream signaling pathways via integrin engagement (e.g., with ITGB1), leading to this favorable transcriptional response—a mechanism requiring future validation through protein phosphorylation assay[37]. Comparison of *TGF-β1* secretion (ELISA) and gene expression (RT-qPCR) revealed a time-dependent correspondence. While both showed an increasing trend at 24 h, only the 48-h time point exhibited statistically consistent elevation in the G-PDA-PDMS group. The weaker correlation at 24 h may be attributed to early protease activation triggered by external stimuli, which could lead to transient, non-specific detection of *TGF-β1* [38]. At 36 h, neither protein secretion nor mRNA expression differed significantly between PDA-PDMS and G-PDA-PDMS. This temporary plateau may reflect a ligand-induced negative-feedback mechanism, wherein *TGF-β1* rapidly induces its own inhibitor Smad7, thereby simultaneously suppressing further transcription and translation [39,40].

This study has several limitations that outline directions for future work. First, wound healing is a complex process involving multiple cell types. Our in vitro model utilized only fibroblasts; future studies should investigate the material’s effects on keratinocytes (for re-epithelialization), endothelial cells (for angiogenesis), and immune cells (e.g., macrophages for immunomodulation). Second, all findings are derived from in vitro experiments. The material’s actual healing efficacy, in vivo biocompatibility, and the long-term stability of the coating under physiological conditions must be rigorously evaluated using appropriate animal wound models. Finally, the precise intracellular signaling mechanism triggered by the modified surface merits deeper exploration.

## Conclusion

In this study, a gelatin-grafted bioactive interface (G-PDA-PDMS) was successfully constructed on the polydimethylsiloxane surface via polydopamine self-polymerization and carbodiimide chemistry. The results of surface characterization and in vitro cell experiments indicate that the PDA-mediated gelatin surface modification not only effectively ameliorates the inherent hydrophobicity of PDMS but also confers bioactive properties that actively regulate cellular behavior and promote extracellular matrix synthesis. This work provides an experimental basis for the development of PDMS-based wound dressings or tissue engineering scaffolds with pro-healing functionalities.
